# The Role of Single Nucleotide Polymorphisms of Monoamine Oxidase B, Dopamine D2 Receptor, and DOPA Decarboxylase Receptors Among Patients Treated for Parkinson’s Disease

**DOI:** 10.1007/s12031-022-01966-3

**Published:** 2022-01-19

**Authors:** Barbara Zapała, Tomasz Stefura, Monika Piwowar, Sylwia Czekalska, Magdalena Zawada, Maria Hadasik, Bogdan Solnica, Monika Rudzińska-Bar

**Affiliations:** 1 Department of Clinical Biochemistry, Jagiellonian University Medical College, Krakow, Poland; 2 Jagiellonian University Medical College, Krakow, Poland; 3 Department of Bioinformatics and Telemedicine, Jagiellonian University Medical College, Krakow, Poland; 4 Department of Hematology Diagnostics and Genetics, The University Hospital, Krakow, Poland; 5 Andrzej Frycz Modrzewski Krakow University, Krakow, Poland

**Keywords:** Parkinson’s disease, Single nucleotide polymorphisms, Receptors

## Abstract

This study aimed to investigate the association between selected variants of genes related to dopamine metabolism pathways and the risk of and progression of Parkinson’s disease (PD). This prospective cohort study was conducted in one academic teaching hospital. The study was conducted on 126 patients diagnosed with idiopathic Parkinson’s disease. Blood samples were collected to conduct a genotyping of MAOB, DRD1, DRD2, and DDC genes. Genotype and allele frequencies of MAOB (rs1799836) variants were not associated with the course of PD. Genotype and allele frequencies of DRD2 (rs2283265) variants were associated with risk of dementia (*p* = 0.001) and resulted in parts II and III of the UPDRS scale (*p* = 0.001). Genotype and allele frequencies of DRD2 (rs1076560) variants were associated with risk of dementia (*p* = 0.001) and resulted in parts II and III of the UPDRS scale (*p* = 0.001). Genotype and allele frequencies of DDC (rs921451) variants were not associated with the course of PD.

## Introduction

Parkinson’s disease (PD) is a chronic and progressive neurodegenerative disorder that affects over 4 million people worldwide (Kalia and Lang 2015). PD is the second, after Alzheimer’s disease, the most common neurodegenerative disorder. Clinically, it is characterized by bradykinesia and at least one of the following symptoms: resting tremor, rigidity, and postural instability (Cacabelos 2017). The research has shown that genetic factors play a pivotal role in developing PD, but especially with youth-onset, but the exact etiology of this disorder is still very elusive (Post et al. 2020). However, the pathogenesis of PD is quite complex and still not fully understood. Previous research suggests that dysfunction of dopaminergic neurotransmission is involved in the pathogenesis of PD (Poewe et al. 2017). Dopaminergic signaling is crucial for motor planning and mental function. Available research suggests the existence of the associations between single nucleotide polymorphisms (SNPs) in different genes involved in neurotransmitter metabolism pathways (Connolly and Lang 2014). However, the exact role of polymorphic variants in dopaminergic neurotransmission regulation is yet to be determined. It is well known that there is a high level of genetic heterogeneity between different populations, and it is necessary to conduct multiple studies to identify the associations between genetic variations in genes that may be related to PD pathophysiology (Pastor 2012).

Monoamine oxidase B (MAOB) is an enzyme-containing flavin-adenine dinucleotide that plays a vital role in the inactivation of neurotransmitters especially by deamination of dopamine. The MAOB gene is located at chromosome Xp11.23. A644G single nucleotide polymorphism (rs1799836), located in exon 13 of the MAOB, is a known functional polymorphism (Kiyohara et al. 2011). A644G was shown to be associated with altered enzyme activity (Liu et al. 2014). The G allele of MAOB A644G polymorphism was associated with lower brain MAOB activity related to PD (Kakinuma et al. 2020).

Dopamine D2 receptor (DRD2) is a G-protein-coupled, and it is mainly expressed in the striatum. The DRD2 gene is located on chromosome 11q23.2. It encodes two molecularly distinct isoforms [the D2 long (D2L) and the D2 short (D2S)] with distinct functions (Gluskin and Mickey 2016; Dubovyk and Manahan-Vaughan 2019). Zhang et al. characterized two SNPs within DRD2, one in intron 5 rs2283265 and the second in intron 6 rs1076560. Both of them were demonstrated to be responsible for splicing. It has been shown that the T allele of both intronic SNPs caused shift splicing from D2S to D2L, which was associated with the changes in glutamate release (Zhang et al. 2007). Previous research has shown an association between these DRD2 polymorphisms and PD phenotypes related to motor fluctuations and dyskinesias (Rieck et al. 2012), whereas the other researchers have shown no associations (Kaiser et al. 2003; Kaplan et al. 2014; Lee et al. 2011). DOPA decarboxylase (DDC) is a pyridoxal 5′-phosphate-reliant enzyme encoded by the DDC gene located on chromosome 7p12.1. This enzyme can facilitate the synthesis of critical neuro-active biogenic amines in the brain (Zhu et al. 2013). Moreover, it was shown that the activity of this enzyme plays a very essential role in central nervous system physiology. Very rare that loss-of-function mutations in the DDC gene lead to a devastating neuro-developmental syndrome known as DDC deficiency (OMIM#608,643). This syndrome, resulting from DDC enzymatic insufficiency, causes severe autonomic, motor, and cognitive impairments which may manifest in early life (Brun et al. 2010). In the context of PD, no studies were showing the association between the genetic variation of the DDC gene with PD development and progression.

This study aimed to investigate the association between selected variants of genes related to dopamine metabolism pathways, representative, functional polymorphisms (rs1799836 of MAOB; rs5326 of DRD1; rs2283265, rs1800497, rs1801028, rs1799732, rs1076560 of DRD2, and rs1065852 of DDC), and the risk of and progression of Parkinson`s disease. The hypothesis was that the specific variants in selected genes involved in the dopamine metabolic pathway were associated with slowing the progression of Parkinson’s disease and improved the effectiveness of levodopa treatment.

## Results

### Study Group

The study was conducted on 126 patients diagnosed with idiopathic Parkinson’s disease. 49 (38.9%) women and 77 (61.1%) men. The median age was 67 years. The control group consisted of 94 individuals (34 women and 60 men) 35–91 years of age. The ethnicity of all patients was Caucasian. In half of the subject’s PD duration was about 9 years. Dementia (MMSE < 24 points) occurred in 23.4% of the patients. The median UPDRS score was 32 points. The complications of levodopa treatment (motor fluctuation, dyskinesia, dystonic movements) were observed in 68.8% of the patients. Deep brain stimulation was applied in 17.5% of the subjects. The distribution of selected characteristics among PD patients is shown in Table 1.

**Table 1:** Characteristics of the study group (*n* = 126)

	**% **(***n***)	***Me***	***IQR***	***Min***	***Max***
Gender					
Women	38.9% (49)	-	-	-	-
Men	61.1% (77)	-	-	-	-
Age (years)	-	67.0	13.0	39.0	95.0
Duration of Parkinson’s disease (years)	-	9.0	8.0	1.0	29.0
Form of Parkinson’s disease					
With dementia	23.4% (29)	-	-	-	-
Without dementia	76.6% (95)	-	-	-	-
UPDRS score (points)	-	32.0	27.0	5.0	94.0
Incidence of treatment complications	68.8% (86)	-	-	-	-
Application of deep brain stimulation	17.5% (22)	-	-	-	-

### Genotype and Allele Frequencies of MAOB (rs1799836) Variant

First, we genotyped the MAOB (rs1799836) SNP in all DNA samples using the TaqMan assay, achieving a success rate of 100%. About 42.3% of the subjects had T/T genotype, 39.0% had C/C genotype, and 18.7% had T/C genotype within the *rs1799836* polymorphism of the MAOB gene. In this case, we did not observe any significant differences in the frequency distribution of *rs1799836* between the total case and control groups. There was no significant relationship between genotype variant and incidence of dementia, motor fluctuations, and application of deep brain stimulation in patients: *p* = 0.116, *p* = 0.497, and *p* = 0.654, respectively. The *p*-value for the relationship between genotype variant and patients’ results in parts II and III of the UPDRS scale was 0.326.

### Genotype and Allele Frequencies of DRD2 (rs2283265) Variant

The vast majority of patients with PD (75.0%) had A/A genotype, 13.3% had C/C genotype, and 11.7% had A/C genotype within the *rs2283265* polymorphism of DRD2 gene (Table 2). There was a significant relationship between genotype variants and incidence of dementia (*p* = 0.001). The mean ± SD age of disease onset in patients with dementia was 57 ± 11, while in patients without dementia, it was 55 ± 12. The mean ± SD duration of the disease in the PD subjects with dementia was 18.3 ± 5.8, while in patients without dementia, it was 13.4 ± 4.7. We excluded the patients with dementia with Lewy bodies from the statistical analyses when correlating *rs2283265* frequency with dementia. The mean ± SD score in MMSE in patients with dementia was 19.1 ± 6.8, while in patients without dementia, it was 25.6 ± 2.8. All the patients with PD without or with dementia met the clinical diagnostic criteria for PD. There was no significant association between *rs2283265* variant and treatment complications or application of deep brain stimulation: *p* = 0.532 and *p* = 0.105, respectively. Only 3.4% of the patients with A/A genotype had dementia. On the other hand, all the patients with C/C genotype had Parkinson’s disease with dementia, and 71.4% of the patients with the A/C genotype had dementia. The relationship between genotype variant and patients’ results in parts II and III of the UPDRS scale was found (*p* = 0.001) (Table 3). Significant differences were found between A/A and C/C genotypes (Table 4). The median score of the patients with A/A genotype was 29 points, and that of the patients with C/C genotype was 50.5 points in parts II and III of the UPDRS scale. Moreover, a difference between A/A and A/C genotypes was observed. Half of the patients with A/A genotype obtained 29 points in parts II and III of the UPDRS scale, while half of those with A/C genotype obtained 64 points. There were no statistical differences (*χ*^2^ = 1263; *df* = 2; *p* = 0,532) when correlating the *rs2283265* with the effectiveness of levodopa treatment.

**Table 2:** Evaluation of the relationship between the genotype variant within the *rs2283265* polymorphism of DRD2 gene and incidence of dementia in patients (*n* = 120)

			Genotype			Test result
			A/A	C/C	A/C	
Form of Parkinson’s disease	With dementia	*N*	3	16	10	*χ*^2^ = *86.954* *df* *= *2 ***p*** = ***0.001***
		%	3.4%	100.0%	71.4%	
	Without dementia	*N*	85	0	4	
		%	96.6%	0.0%	28.6%	

*χ*^2^ statistic, *df* degrees of freedom, *p* statistical significance

**Table 3:** Evaluation of the relationship between the genotype variant within the *rs2283265* polymorphism of *DRD2* gene and patients’ results in parts II and III of the UPDRS scale (*n* = 120)

	Genotype	***χ***^2^	***df***	***p***	***Min***	***Max***	***Me***
UPDRS score (points)	A/A	48.51	2	< 0.001	5.00	84.00	29.00
	C/C				33.00	94.00	50.50
	A/C				32.00	92.00	64.00

*χ*^2^ test statistic, *df* degrees of freedom, *p* statistical significance. *Min* minimum, *Max* maximum, *Me* median

**Table 4:** Evaluation of differences in patients’ results in parts II and III of the UPDRS scale between genotypes within the *rs2283265* polymorphism of *DRD2* gene (*n* = 120)

	Genotype		***p***	
UPDRS score (points)	A/A	C/C	< 0.001	***
	A/A	A/C	< 0.001	***
	C/C	A/C	0.358	

*p* statistical significance

*p < 0.05; **p < 0.01; ***p < 0.001

### Genotype and Allele Frequencies of DRD2 (rs1076560) Variant

Overall, 49.6% of the subjects had an A/C genotype. Almost the same percentage of patients (48.8%) had C/C genotype, while only 1.6% had A/A genotype within the *rs1076560* polymorphism of DRD2 gene (Table 5). There was a relationship between genotype variants and incidence of dementia (*p* < 0.001). For the other tested variables — motor fluctuations and application of deep brain stimulation, the *p*-values were 0.655 and 0.605, respectively. None of the patients with the A/A genotype had dementia. In the A/C genotype group patients, 45.0% had a form of PD with dementia. Among the patients with C/C genotype, only 3.4% had dementia. Due to the insufficient number of subjects with A/A genotype (2), it was necessary to exclude them from assessing the relationship between genotype variants and the results of the UPDRS scale. There was a relationship between genotype variants and patients’ results in parts II and III of the UPDRS scale for A/C and C/C genotypes (*p* < 0.001). The average results of the patients with A/C and C/C genotypes in this scale were: 43.3 and 26.2 points, respectively (Table 6). The statistical significant differences were not observed (*χ*^2^ = 0.845; *df* = 2; *p* = 0.655) when correlating the frequency of *rs1076560* with the effectiveness of levodopa treatment.

**Table 5:** Evaluation of the relationship between the genotype variant within the *rs1076560* polymorphism of *DRD2* gene and incidence of dementia in patients (*n* = 123)

			Genotype			Test result
			A/A	C/C	A/C	
Form of Parkinson’s disease	With dementia	N	0	27	2	χ2 = 28.905 df = 2 *p* = 0.001
		%	0.0%	45.0%	3.4%	
	Without dementia	N	2	33	57	
		%	100.0%	55.0%	96.6%	

*χ*^2^ test statistic, *df* degrees of freedom, *p* statistical significance

**Table 6:** Evaluation of the relationship between the genotype variant in the *rs1076560* polymorphism of the *DRD2* gene and patients’ results in parts II and III of the UPDRS scale (*n* = 123)

					Descriptive statistics	
		***t***	***df***	***p***	***M***	***SD***
Genotype	UPRDS score (points)	−5.16	98	< 0.001		
	A/C				43.28	22.10
	C/C				26.20	13.18

*t* test statistic, *df* degrees of freedom, *p* statistical significance, *M* median *SD* standard deviation

### Genotype and Allele Frequencies of DDC (rs921451)

Overall, 48.4% of the patients had C/T genotype, 33.0% had T/T genotype, and 18.7% had C/C genotype within the *rs921451* polymorphism of the DDC gene. There was no significant relationship between genotype variant and incidence of dementia, motor fluctuations, and application of deep brain stimulation: *p* = 0.796, *p* = 0.205, and *p* = 0.394, respectively. The *p*-value for the relationship between genotype variant and patients’ results in parts II and III of the UPDRS scale was 0.239. We did not observe the statistical significant correlation (*χ*^2^ = 3166; *df* = 2; *p* = 0.205) between the *rs921451* and the side effects of levodopa treatment.

## Discussion

This study investigated the associations between polymorphisms within the MAOB (*rs1799836*), DRD2 (*rs228365* and *rs1076560*), DDC (*rs921451*) genes and phenotypes in PD patients treated with levodopa. We hypothesized that the selected polymorphisms are associated with the progression of the PD and levodopa treatment complication. Understanding the etiology of PD and providing a successful treatment remains a challenge. Identifying potential predictors of the treatment outcomes paves the way for more personalization and an individual approach to the treatment. The MAOB *rs1799836* variant was not associated with the PD phenotype. We did not find any statistically significant associations of MAOB variant with the occurrence of the PD in the Polish population. There was no significant relationship between motor fluctuation, side-effects of the levodopa treatment, the onset of dementia or deep-brain stimulation treatment, and MAOB polymorphism.

Kiyohara et al. reported that polymorphism *rs1799836* of the MAOB gene might play an important role in PD susceptibility in the Japanese population (Kiyohara et al. 2011). This association was later confirmed in a meta-analysis by Sun et al. published in 2016 (Sun et al. 2014). Hao et al. verified the genotype frequencies of MAO-B *rs1799836* A/AA, AG, G/GG in the Chinese PD patients’ population, 74.4%, 14.1%, and 11.5%, respectively (Hao et al. 2015). A study by Moreau et al. showed no significant associations between the response to L-dopa during PD treatment and MAOB *rs1799836* variant (Moreau et al. 2015). A more recent study identified the rs1799836 polymorphism as a potential predictor of putaminal dopamine turnover in early PD.

Additionally, the MAOB TT allele was linked to high enzyme activity leading to higher intrinsic dopamine turnover, which has been demonstrated to constitute a risk factor for motor complications (Löhle et al. 2018). Sampaio et al. reported that patients carrying MAO-B (rs1799836) A and AA genotypes are prone to levodopa-induced-dyskinesia (Sampaio et al. 2018).

Presented results demonstrate that the CC genotype of DRD2 *rs228365* was associated with dementia compared to the AA genotype. The study also suggests the CC genotype of DRD2 *rs228365* as a genetic marker of more severe impairments in PD patients. The patients with this genotype had the UPDRS part II and III score of above 50 compared to the AA genotype (with a UPDRS part II and III score of below 29). The AA genotype was associated with a lower progression of PD. There are very few studies investigating the role of DRD2 *rs228365* polymorphism in patients suffering from PD. There was no correlation between the *rs2283265* polymorphism in the DRD2 gene and levodopa treatment and the need for deep-brain stimulation treatment. Authors of similar studies have shown the effect of *rs2283265* on the treatment of PD patients as they were able to demonstrate that the polymorphism influenced the development of dyskinesias.

According to the results obtained by Rieck et al., a haplotype (TTCTA) derived from *rs2283265* polymorphism in the DRD2 gene region is associated with dyskinesia during levodopa treatment, which results from the reduced expression of the DRD2 gene (Rieck et al. 2012). This results from the inhibition of negative feedback and lower control over dopamine release. Masellis et al. reported that single nucleotide polymorphisms *rs2283265* in the DRD2 were found to be significantly associated with a favorable peak response to rasagiline at 12 weeks in early Parkinson’s disease (Masellis et al. 2016). Previous studies also identified associations between this polymorphism, other genetic variants, and severe cocaine abuse (Sullivan et al. 2013; Stolf et al. 2019). There are also reports highlighting the potential role of this polymorphism in developing various psychiatric disorders, including schizophrenia, ADHD, or others from the autism spectrum (Glatt et al. 2009; Gadow et al. 2014).

According to our results, the A/C genotype in *rs1076560* polymorphism of the DRD2 gene is associated with dementia. It is essential to mention that the AA genotype was underrepresented in our study group. The patients with the A/C genotype had also significantly higher mean scores in parts II and III of the UPDRS scale.

An article by Miller et al. presented results suggesting a role of *rs1076560* DRD2 polymorphism in predicting Parkinson’s disease gait impairment and medication responsiveness of specific gait functions. The authors explained that observed outcomes might result from reduced striatal D2 receptor expression (T allele carriers), which induces gait dysfunction compared to homozygous patients for the G allele (Miller et al. 2018). Masellis et al. identified rs1076560 polymorphism in the gene DRD2 (besides *rs2283265*) as associated with the peak clinical response to rasagiline (Masellis et al. 2016).

The DDC *rs921451* variant was not associated with the PD phenotype. There were no statistically significant associations between DDC variants and PD treatment in the studied group. Also, DDC *rs921451* polymorphism did not influence side-effects of the levodopa treatment, the onset of dementia during PD, or deep-brain stimulation treatment.

The *rs921451* variant of the DDC gene was reported by Devos et al. to influence the motor response to L-dopa but do not significantly change peripheral pharmacokinetic parameters for L-dopa and dopamine (Devos et al. 2014). However, results reported by Moreau et al. presented no significant association between the *rs921451* variant of the DDC gene and the response to L-dopa (Moreau et al. 2015). When the role of the *rs921451* polymorphism of the DDC in striatal dopamine turnover in de novo PD was investigated, the authors did not report significant findings (Löhle et al. 2018). Publication by Redenšek et al. reported that carriers of at least one DDC *rs921451* C allele had higher odds for developing orthostatic hypotension during dopaminergic treatment of the PD (Redenšek et al. 2019).

There are several limitations to this study. It was conducted in one center and included patients from one country. The study group was relatively homogenous in terms of the ethnicity of patients. Therefore, the generalizability of the results obtained is yet to be determined. Additionally, PD includes multiple types and subtypes. Their division is based on scoring patterns for baseline motor, cognitive, and psychiatric measures. Unfortunately, we could not recruit patients with different types of PD to the study group. It may further limit the generalizability of the results over differently manifested types of PD. There is a risk that investigated polymorphisms play a different role in different clinical manifestations.

## Materials and Methods

### Study Design

This prospective cohort study was conducted in one academic hospital between November 2018 and November 2020. A neurologist expert of movement disorders identified all the patients with PD (based on the UK Parkinson’s Disease Society Brain Bank Criteria for clinically probable disease). All the PD patients underwent the following clinical evaluation: Mini-Mental State Examination (MMSE), motor examination according to the Unified Parkinson’s Disease Rating Scale (UPDRS, part II and part III), and Hoehn and Yahr Staging (H&Y). ON–OFF motor fluctuations, dyskinesia, dystonia, off stage were monitored using the patients’ diary. All the subjects were invited to provide a blood sample for genetic assessment. The patients in the control group had no previous diagnosis of neurodegenerative or malignant disease or family history of these disorders.

### TaqMan SNP Genotyping

From all the individuals, we collected blood samples using EDTA tubes. Genomic DNA (gDNA) was extracted from whole blood using silica-based spin columns such as the QIAam DNA Blood Mini Kit from Qiagen (Valencia, CA). The quality of gDNA was assessed using NanoDrop ND-1000 spectrophotometer from NanoDrop Technologies (Wilmington, DE, USA), and only high-quality gDNA was used for genotyping. DNA samples were then stored at –20 °C until genotype analysis. The genotyping was carried out with ready-to-order Custom TaqMan SNP genotyping assays (from Applied Biosystems, Foster City, CA). We performed the genotyping of only the key SNPs utilized to identify MAOB (rs1799836, assay ID C__8878790_10), DRD1 (rs4532, C__1011777_10), DRD2 (rs2283265, assay ID C__16070796_10, rs1800497, C__7486676_10, rs1801028, C__10725_20, rs1799732, C__33641686_10, rs1076560, assay ID C__2278888_10) and DDC (rs1065852, assay ID C__8320238_10; from Applied Biosystems). The assay used in the study included forward and reverse PCR primers for selected SNPs and two differently labeled TaqMan minor groove binder (MGB) probes. The biallelic SNP was located in the middle third of the probe. Each allele-specific MGB probe was labeled with a fluorescent reporter dye (either a FAM or a VIC reporter molecule) and was attached to a fluorescence quencher. For the intact MGB probe, the reporter dye was quenched. We used 20 ng of gDNA for amplification as per manufacturer’s directions scaled to a total volume of 25 µl. During PCR, the 50-nuclease activity of Taq DNA polymerase cleaved the reporter dye (FAM or VIC) from an MGB probe that was completely hybridized to the DNA strand. The PCR was performed according to the manufacturer’s instructions provided by Applied Biosystems. The PCR thermal cycling was as follows: initial denaturing at 95 °C for 30 s; 40 cycles of 92 °C for 5 s and 60 °C for 20 s. Post-amplification products were analyzed on an Applied Biosystems ViiA 7 Real-Time PCR System, and genotype calls were determined manually by a comparison to no template controls. An increase in either FAM or VIC dye fluorescence indicated homozygosity for FAM- or VIC-specific alleles (X:X or Y:Y), and an increase in the fluorescence of both dyes indicated heterozygosity (X:Y). The two colors (FAM or VIC) were detected using ViiA 7 Realt-Time PCR System. Genotype calls were assessed with Applied Biosystems TaqMan Genotyper Software.

### Statistical Analyses

Data were analyzed using the Statistica software version 10. The genotype distribution and allele frequency of selected SNPs of MAOB, DRD1, DRD2, and DDC gene in the control and PD group were compared using chi-squared test. Bonferroni’s post hoc test was used to determine the differences between the groups. We assessed Hardy–Weinberg equilibrium via a goodness-of-fit *χ*^2^-test Kruskal–Wallis test R to compare the observed and expected genotype results in examined groups. A *p*-value ≤ 0.005 was considered statistically significant. At first, the genotypic and allelic distribution in both (study and control group) were analyzed, and only the statistically significant results were selected to the further analysis in which the genotypic and allelic distribution in the group of Parkinson disease patients with different disease progression and with or without levodopa-induced dyskinesia were analyzed.

## Conclusions

Our data provide a solid from which we can work towards the personalization of PD treatment since it may help identify fragile PD patients who would benefit from a less aggressive dopaminergic treatment. All of the receptors are important and involved in the dopamine pathway. Further studies with a larger population will help clarify the interpretation of these data.

## Data Availability

Data will be made available upon reasonable request.
